# Sequencing of the complete mitochondrial genome of a fish-parasitic flatworm *Paratetraonchoides inermis* (Platyhelminthes: Monogenea): tRNA gene arrangement reshuffling and implications for phylogeny

**DOI:** 10.1186/s13071-017-2404-1

**Published:** 2017-10-10

**Authors:** Dong Zhang, Hong Zou, Shan G. Wu, Ming Li, Ivan Jakovlić, Jin Zhang, Rong Chen, Gui T. Wang, Wen X. Li

**Affiliations:** 1 Key Laboratory of Aquaculture Disease Control, Ministry of Agriculture, and State Key Laboratory of Freshwater Ecology and Biotechnology, Institute of Hydrobiology, Chinese Academy of Sciences, Wuhan, People’s Republic of China; 2 University of Chinese Academy of Sciences, Beijing, People’s Republic of China; 3 Bio-Transduction Lab, Wuhan Institute of Biotechnology, Wuhan, People’s Republic of China

**Keywords:** Mitochondrial genome, Tetraonchidea, Neodermata, Monopisthocotylea, A + T bias, Phylogenomics, Morphology, Gene order

## Abstract

**Background:**

*Paratetraonchoides inermis* (Monogenea: Tetraonchoididae) is a flatworm parasitising the gills of uranoscopid fishes. Its morphological characteristics are ambiguous, and molecular data have never been used to study its phylogenetic relationships, which makes its taxonomic classification controversial. Also, several decades of unsuccessful attempts to resolve the relationships within the Monogenea present a strong indication that morphological datasets may not be robust enough to be used to infer evolutionary histories. As the use of molecular data is currently severely limited by their scarcity, we have sequenced and characterized the complete mitochondrial (mt) genome of *P. inermis*. To investigate its phylogenetic position, we performed phylogenetic analyses using Bayesian inference and maximum likelihood approaches using concatenated amino acid sequences of all 12 protein-coding genes on a dataset containing all available monogenean mt genomes.

**Results:**

The circular mt genome of *P. inermis* (14,654 bp) contains the standard 36 genes: 22 tRNAs, two rRNAs, 12 protein-encoding genes (PCGs; *Atp8* is missing) and a major non-coding region (mNCR). All genes are transcribed from the same strand. The A + T content of the whole genome (82.6%), as well as its elements, is the highest reported among the monogeneans thus far. Three tRNA-like cloverleaf structures were found in mNCR. Several results of the phylogenomic analysis are in disagreement with previously proposed relationships: instead of being closely related to the Gyrodactylidea, Tetraonchidea exhibit a phylogenetic affinity with the Dactylogyridea + Capsalidea clade; and the order Capsalidea is neither basal within the subclass Monopisthocotylea, nor groups with the Gyrodactylidea, but instead forms a sister clade with the Dactylogyridea. The mt genome of *P. inermis* exhibits a unique gene order, with an extensive reorganization of tRNAs. Monogenea exhibit exceptional gene order plasticity within the Neodermata.

**Conclusions:**

This study shows that gene order within monopisthocotylid mt genomes is evolving at uneven rates, which creates misleading evolutionary signals. Furthermore, our results indicate that all previous attempts to resolve the evolutionary history of the Monogenea may have produced at least partially erroneous relationships. This further corroborates the necessity to generate more molecular data for this group of parasitic animals.

**Electronic supplementary material:**

The online version of this article (10.1186/s13071-017-2404-1) contains supplementary material, which is available to authorized users.

## Background

Classification and phylogeny of the Monogenea have been debated for decades without reaching a consensus. Therefore, several classification systems, based on different morphological characters, coexist (e.g. [1–7]). These often use inconsistent terminology; e.g. Monogenea vs Monogenoidea, Monopisthocotylea vs Polyonchoinea, etc. (for further details see Table 1 in Mollaret et al. [8]). To simplify comparison with previous studies and discussion of our results, we follow the naming system of Boeger & Kritsky [4], with four exceptions: we use order rank for Tetraonchidea (which was split into two parts by Boeger & Kritsky: Tetraonchidae and Amphibdellatidae were assigned to Dactylogyridea, and the remaining Bothitrematidae and Tetraonchoididae were assigned to Gyrodactylidea), Monogenea instead of Monogenoidea, Monopisthocotylea instead of Polyonchoinea, and Polyopisthocotylea instead of Oligonchoinea.

**Table 1:** Annotated mitochondrial genome of *Paratetraonchoides inermis*

Gene	Position		Size	Intergenic nucleotides	Codon		Anti-codon
	From	To			Start	Stop	
*cox*3	1	651	651		ATG	TAA	
*trnH*	652	716	65				GTG
*cytb*	717	1820	1104		ATG	TAA	
*nad4L*	1825	2091	267	4	ATG	TAG	
*nad*4	2058	3272	1215	-34	ATG	TAA	
*trnQ*	3274	3336	63	1			TTG
*trnF*	3336	3400	65	-1			GAA
*trnM*	3394	3457	64	-7			CAT
*atp*6	3458	3973	516		ATG	TAA	
*nad*2	3990	4832	843	16	ATG	TAA	
*trnA*	4853	4920	68	20			TGC
*trnD*	4983	5050	68	62			GTC
*nad*1	5081	5977	897	30	ATG	TAA	
*trnI*	5989	6056	68	11			GAT
*trnK*	6059	6130	72	2			CTT
*nad*3	6131	6481	351		ATG	TAA	
*trnW*	6497	6562	66	15			TCA
*trnN*	6603	6670	68	40			GTT
*trnP*	6680	6743	64	9			TGG
*trnS*	6789	6849	61	45			GCT
*cox*1	6882	8474	1593	32	GTG	TAA	
*trnT*	8460	8523	64	-15			TGT
*rrnL*	8524	9460	937				
*rrnS*	9473	10,178	706	12			
*cox*2	10,192	10,764	573	13	ATG	TAA	
*trnE*	10,764	10,833	70	-1			TTC
*nad*6	10,837	11,283	447	3	ATG	TAA	
*trnL*	11,325	11,387	63	41			TAA
*trnY*	11,421	11,483	63	33			GTA
*trnL*	11,482	11,544	63	-2			TAG
*trnV*	11,545	11,611	67				TAC
*trnG*	11,612	11,673	62				TCC
*trnS*	11,686	11,753	68	12			TGA
*trnC*	11,765	11,823	59	11			GCA
*trnR*	11,836	11,896	61	12			TCG
*nad*5	11,915	13,453	1539	18	ATG	TAA	

*Paratetraonchoides inermis* Bychowsky, Gussev & Nagibina, 1965 (Monogenea: Tetraonchoididae) is a parasitic flatworm usually found on gills of fishes belonging to the family Uranoscopidae. It was originally assigned to the Tetraonchoididae (Tetraonchidea) based on several morphological characteristics: a considerably extended ribbon-shaped body, absence of eyes and middle hooks, and a digestive system typical for this family [9]. However, as the parasite uses 16 marginal gyrodactylid-type (hinged) edge hooks to attach itself to the gills of its host, later it was reassigned to Gyrodactylidea by Boeger & Kritsky [4]. Concerning relationships among the orders Dactylogyridea, Tetraonchidea, Capsalidea and Gyrodactylidea (DTCG group henceforth), Bychowsky [1] argued that the ancestors of the Tetraonchidea were morphologically closer to the Gyrodactylidea than to the Dactylogyridea (note that capsalids were assigned to the Dactylogyridea in this classification). In contrast, in taxonomic classification presented by Lebedev [2] includes the Tetraonchidea, together with the Dactylogyridea and Capsalidea, into the superorder Dactylogyria, whereas the Gyrodactylidea was elevated to the superorder level (Gyrodactylia). This classification was partly supported by Justine [5], based on spermatology data: sperm pattern four was found in the orders Tetraonchidea and Dactylogyridea, whereas sperm patterns 2a and 2c were found in the Capsalidea and Gyrodactylidea, respectively. The fact that these traits produce incongruent results, and that several decades of research have failed to produce a consensus on the phylogeny of DTCG group, presents a strong indication that morphological datasets may not provide a sufficiently robust method to establish a comprehensive phylogenetic hypothesis for DTCG group and the Monogenea.

With rapid advances in sequencing techniques, accompanied by exponentially increasing the availability of molecular data, molecular phylogenetics is becoming the tool of choice for resolving the evolutionary relationships within this group of animals [10, 11]. Despite this, the availability of molecular data for DTCG remains limited and unbalanced (some orders are underrepresented) to infer their phylogenetic relationships with high resolution. For example, currently (July 2017), only 12 molecular records are available in the GenBank database for the Tetraonchidea. The only molecular marker used so far to study the phylogenetics of DTCG was 18S ribosomal RNA [11]. This marker produced a topology somewhat similar to Justine’s results [5]: (Dactylogyridea + Tetraonchidea) + (Monocotylidea + (Capsalidea + Gyrodactylidea)). However, as single-gene markers may not carry a sufficient amount of information to provide high phylogenetic resolution, future studies shall probably increasingly rely on much more powerful phylogenomic approaches [12, 13].

Owing to the abundance of mitochondria in animal tissues, maternal inheritance, the absence of introns, the small size of genomes in metazoans, and an increasingly large set of available orthologous sequences, metazoan mitochondrial (mt) genomes have become a popular tool in population genetics [14], phylogenetics [15, 16] and diagnostics [12]. Comparisons of the arrangements of genes have garnered much scientific attention, as they carry a wealth of complementary resources with potential applications in molecular systematics [17, 18]. Although gene order within neodermatan (i.e. parasitic flatworms) mt genomes is assumed to be remarkably conserved [16, 19, 20], several exceptions have been reported, including African/Indian schistosomes [19, 21], polyopisthocotylids [16, 22, 23] and a single monopisthocotylid [24].

Here, we sequenced, annotated and characterized the first tetraonchid mt genome sequence, belonging to *P. inermis*. We compared it structurally to all available (July 2017) neodermatan mt genomes and used all available monogenean mt genome sequences to reconstruct the phylogenetic relationships within the entire class.

## Methods

### Specimen collection and DNA extraction

Monogeneans were collected on 27th September 2016 from the gills of *Ichthyscopus lebeck* (Bloch & Schneider, 1801) obtained from Dong-He market in Zhoushan, Zhejiang Province, China (29°56′–40°62′N; 122°18′–12°30′E). *Paratetraonchoides inermis* was identified morphologically by the hard parts of the haptor (dorsal and ventral connective bars, marginal hooks) and reproductive organs (male copulatory organ and vaginal armament) [9] under a stereomicroscope and a light microscope. The parasites were preserved in 99% ethanol and stored at 4 °C. Total genomic DNA was extracted from about 120 entire parasites using the TIANamp Micro DNA Kit (Tiangen Biotech, Beijing, China), according to the manufacturer’s recommended protocol, and stored at -20 °C.

### Amplification and sequencing

Conducted as described before [25], with minor modifications: partial sequences of *nad*5*, nad*1*, cox*1*, cox*3*, cox*2 and *rrnS* genes were amplified by PCR using six degenerate primer pairs (Additional file 1). Based on these newly sequenced fragments, we designed specific primers for amplification and sequencing of the whole mitogenome (Additional file 1: Table S1). PCR was performed in a 20 μl reaction mixture, containing 7.4 μl dd H_2_O, 10 μl 2× PCR buffer (Mg^2+^, dNTP plus, Takara, Dalian, China), 0.6 μl of each primer, 0.4 μl r*Taq* polymerase (250 U, Takara), and 1 μl of DNA template. Amplification was conducted under the following conditions: initial denaturation at 98 °C for 2 min, followed by 40 cycles at 98 °C for 10 s, 48–60 °C for 15 s, 68 °C for 1 min/kb, and a final extension at 68 °C for 10 min. PCR products were sequenced bi-directionally at Sangon Company (Shanghai, China) using the primer-walking strategy.

### Sequence annotation and analyses

After BLASTn analysis [26], the mitochondrial genomic sequence was assembled manually in a stepwise manner. The mt genome was aligned against the mt genomic sequences of other published monogeneans using MAFFT 7.149 [27] to determine approximate gene boundaries. The annotation was further fine-tuned using Geneious [28] adopting one capsalid mt genome, *Neobenedenia melleni* (MacCallum, 1927) (JQ038228) as the reference, and finally recorded in a Word document. Protein-coding genes (PCGs) were found by searching for ORFs (employing genetic code 9, echinoderm mitochondrial) and checking nucleotide alignments against the reference genome in Geneious. All tRNAs were identified using ARWEN [29], DOGMA [30], and MITOS [31] web servers. The two rRNAs, *rrnL* and *rrnS*, were also preliminarily found using MITOS, and their precise boundaries determined by alignment with closely related orthologs in Geneious. The NCBI submission file and tables with statistics for mt genomes were created using a home-made GUI-based program, MitoTool [32]. A nucleotide composition table was then used to make the broken line graph of skewness and A + T content in ggplot2 [33]. Codon usage and relative synonymous codon usage (RSCU) for 12 protein-encoding genes (PCGs) of seven analyzed monopisthocotylids were initially computed with MEGA 5 [34], then further sorted using custom-made Python scripts [35], and finally imported into ggplot2 to draw the RSCU figure. Rearrangement events in studied mt genomes and pairwise comparisons of gene orders of seven monogeneans were analyzed with CREx web tool [36] using the common interval measurement.

### Phylogenetic analyses

Phylogenetic analyses were conducted using amino acid sequences of PCGs of the newly sequenced mt genome (*P. inermis*) and all 17 monogenean mt genomes available in the GenBank (Additional file 2: Table S2). Two species of the order Tricladida, *Crenobia alpina* (Dana, 1766) (KP208776) and *Obama* sp. MAP-2014 (NC_026978), were used as outgroups, as suggested in our previous study [25]. A fasta file with nucleotide sequences for all 12 PCGs was extracted from GenBank files and translated into amino acid sequences (employing genetic code 9, echinoderm mitochondrial) using MitoTool. All genes were aligned in batches with MAFFT, integrated into another GUI-based program written by us, BioSuite [37]. BioSuite was also used to concatenate these alignments and generate phylip and nexus format files. Phylogenetic analyses were conducted using maximum likelihood (ML) and Bayesian inference (BI) methods. Selection of the most appropriate evolutionary model for the dataset was carried out using ProtTest [38]. Based on the Akaike information criterion, MtArt + I + G + F was chosen as the optimal model for ML analysis, and LG + I + G + F for the BI analysis. ML analysis was performed in RaxML GUI [39] using a ML + rapid bootstrap (BP) algorithm with 1000 replicates. BI analysis was performed in MrBayes 3.2.6 [40] with default settings, and 5 × 10^6^ metropolis-coupled MCMC generations. Finally, phylograms and gene orders were visualized and annotated by iTOL [41] with the help of several dataset files generated by MitoTool.

## Results and discussion

### Genome organization and base composition

**Fig. 1:**
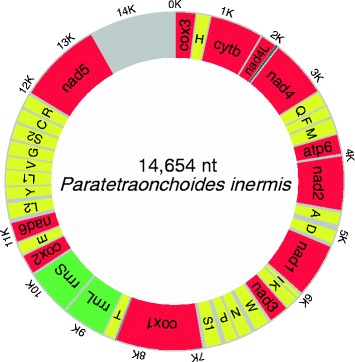
Visual representation of the circular mitochondrial genome of *Paratetraonchoides inermis*. Protein-coding genes (12) are red, tRNAs (22) are yellow, rRNAs (2) are green, and non-coding regions are grey

**Fig. 2:**
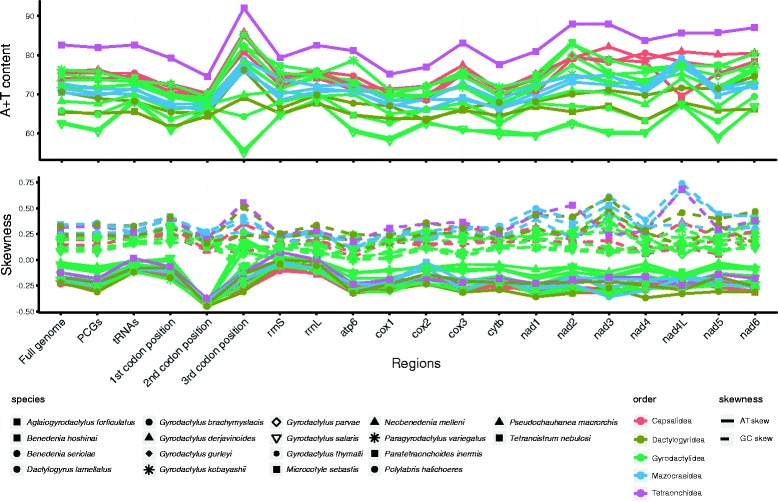
A + T content and skewness of individual elements and the complete genome. Species are coloured according to their taxonomic placement at the order level

The circular mitochondrial genome of *P. inermis* is 14,654 bp in size (GenBank accession number KY856918). The mitogenome is comprised of 12 protein-encoding genes (PCGs), 22 tRNA genes, two rRNA genes, and a major non-coding region (mNCR) (Fig. 1). The genome lacks the *Atp8* gene, which is common for flatworms [42]. All genes are transcribed from the same strand. Six overlaps and 22 intergenic regions were found in the genome (Table 1). Noteworthy, the A + T content of the whole genome (82.6%), concatenated PCGs (81.9%), concatenated rRNA genes (81.1%), concatenated tRNA genes (82.6%), and even individual elements (three codon positions, two rRNAs, all 12 individual PCGs) of the genome, are the highest reported among the monogenean mitogenomes characterized so far (Additional file 2: Table S2; Fig. 2). Nucleotide skewness of the mt genome (as well as its elements) did not differ from other monogeneans (Fig. 2).

### Protein-coding genes and codon usage

Coalesced PCGs were 9996 bp in size, with a notably high A + T content of 81.9%. This was also reflected in individual PCGs: from 75.1% (*cox*1) to 87.9% (*nad*2 and *nad*3) (Table 2). Apart from *cox*1 (which used GTG), ATG was the initial codon for all other PCGs. Among the terminal codons, 11 out of 12 were TAA, whereas *nad*4L used TAG. No abbreviated stop codons (T--) were found (Table 1).

**Table 2:** Nucleotide composition and skewness of different elements of the studied mitochondrial genome

Regions	Size (bp)	T(U)	C	A	G	AT (%)	GC (%)	AT skew	GC skew
PCG	9996	48.5	6.1	33.4	12	81.9	18.1	-0.185	0.329
1st codon position	3332	42.3	6.2	36.9	14.6	79.2	20.8	-0.067	0.401
2nd codon position	3332	51.3	10.2	23.2	15.3	74.5	25.5	-0.378	0.2
3rd codon position	3332	52	1.8	40	6.2	92	8	-0.13	0.556
*atp*6	513	50.1	7.8	31	11.1	81.1	18.9	-0.236	0.175
*cox*1	1590	44.8	8.6	30.3	16.3	75.1	24.9	-0.194	0.308
*cox*2	570	45.8	7.5	31.1	15.6	76.9	23.1	-0.192	0.348
*cox*3	648	50.5	5.4	32.6	11.6	83.1	17	-0.216	0.364
*cytb*	1101	45.9	8.4	31.7	14.1	77.6	22.5	-0.183	0.255
*nad*1	894	49.7	5.4	31.2	13.8	80.9	19.2	-0.228	0.439
*nad*2	840	52.5	2.9	35.4	9.3	87.9	12.2	-0.195	0.529
*nad*3	348	51.4	4.6	36.5	7.5	87.9	12.1	-0.17	0.238
*nad*4	1212	48.8	5.9	34.9	10.4	83.7	16.3	-0.167	0.279
*nad4L*	264	53.4	2.3	32.2	12.1	85.6	14.4	-0.248	0.684
*nad*5	1536	48.9	5	36.8	9.2	85.7	14.2	-0.14	0.297
*nad*6	444	51.4	4.1	35.6	9	87	13.1	-0.181	0.379
*rrnL*	937	41.1	6.3	41.4	11.2	82.5	17.5	0.004	0.28
*rrnS*	706	36.7	7.8	42.6	12.9	79.3	20.7	0.075	0.247
NCR	1201	45.5	5	40.5	8.9	86	13.9	-0.058	0.281
tRNAs	1432	40.8	6.4	41.8	11	82.6	17.4	0.013	0.269
Full genome	14,654	46.5	6	36.1	11.4	82.6	17.4	-0.126	0.312

*Abbreviations*: *PCG* protein-coding genes, *NCR* non-coding region

**Fig. 3:**
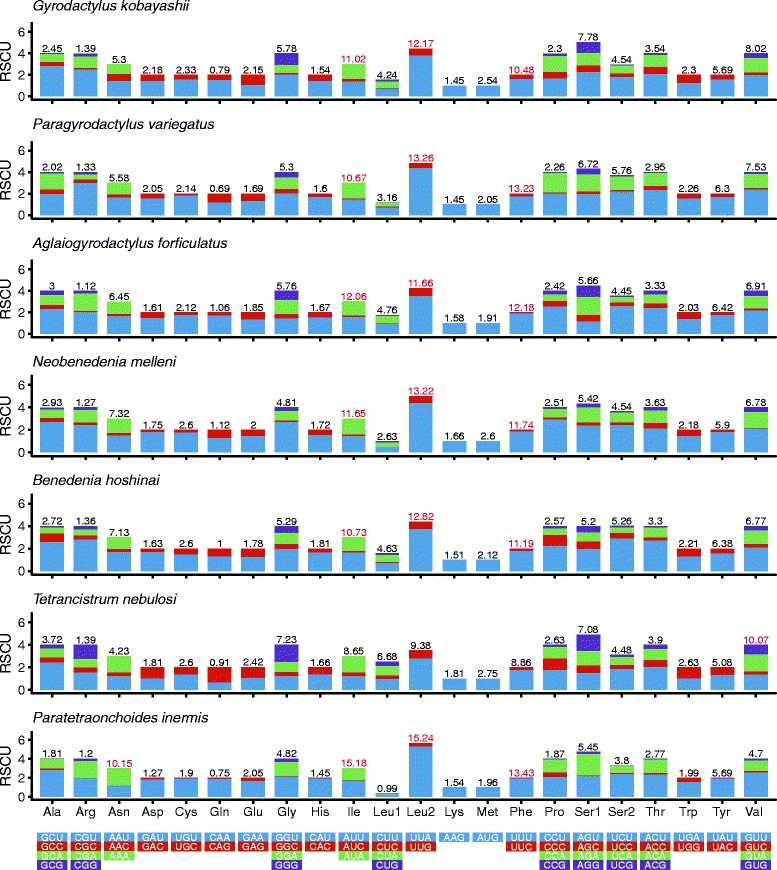
Relative synonymous codon usage (RSCU) of seven monopisthocotylid representatives. Codon families are labelled on the x-axis. Values on the top of the bars denote amino acid usage

Codon usage, RSCU, and codon family proportion (corresponding to the amino acids usage) were investigated among seven monopisthocotylid representatives (Fig. 3). Except for *Tetrancistrum nebulosi* (Young, 1967), the most abundant codon families were Leu2, Ile, and Phe. This is comparable to Lepidoptera [43] and Nemertea [44]. Noteworthy, the studied mt genome exhibited a strong preference for the A + T-rich members of these four codon families (>10%, Phe, Ile, Leu2 and Asn in Fig. 3), whereas three codons mainly composed of G + C (CGC, GCG and CUG) were not found at all. This all corresponds well to the exceptionally high A + T bias of this mt genome. Overall, A + T-rich codons were favored over synonymous codons with lower A + T content among all seven considered monopisthocotylids (Fig. 3). This A + T preference is notably exemplified by the Leu2 family (TTA and TTG), where the TTA codon accounted for 86.92 ± 4.64%.

### Transfer RNA genes

All 22 standard tRNAs were found (Table 1), and most of them exhibited the conventional cloverleaf structure. Exceptions were *trnS1* ^(AGN)^ and *trnC*, which lacked DHU arms. The unorthodox *trnS1* ^(AGN)^ is commonplace among all sequenced monogeneans [12, 15, 16, 22–24, 45–51], and possibly even all flatworms [42]. The unpaired DHU-arm in *tRNA* ^*cys*^ was also reported in most monogeneans apart from *Pseudochauhanea macrorchis*, *M. sebastis*, *Polylabris halichoeres* (Wang & Yang, 1998), *T. nebulosi* and *N. melleni* [16, 22, 23, 45, 52]. A slight preference for the A nucleotide (AT skewness, 0.013) was found in concatenated tRNAs of the *P. inermis* mt genome, which is an exclusive feature among the analyzed monogeneans (Fig. 2), all of which exhibit a preference for the T nucleotide.

### Non-coding regions

The major non-coding region (mNCR), 1201 bp in size and located between *nad*5 and *cox*3, had a slightly higher A + T content (86%) than other parts of the genome (Table 2). Within the mNCR, there were two minor repetitive regions, both consisting of two repeats, 19 and 16 bp in size. Three tRNA-like cloverleaf structures were found in the mNCR (Additional file 3: Figure S1), among which *trnS1*-like and *trnL1*-like sequences contained modified standard anti-codons (ACT and AAG respectively), whereas *trnS2*-like had a standard TAA anticodon. Average sequence similarity values between the three tRNA-like pseudo-genes and the corresponding functional monogenean tRNA homologs were low (41.45 ± 4.61% *trnS1*-like, 37.14 ± 3.84% *trnL1*-like, and 40.32 ± 6.01% *trnS2*-like), which indicates that they may not be functional. Such tRNA-like sequences were also observed in mNCRs of many lepidopteran insects [53, 54]. These three tRNA-like genes could be a remnant of the tandem-duplication-random-loss (TDRL) process, and the associated heightened rates of substitutions and indels in duplicated genes. A similar hypothesis was put forward by Cameron [55] with regard to the presence of tRNA-like sequences in the mNCRs of many lepidopteran insects [53, 54]. However, due to the limited data we have at disposal, functionality and presence of such tRNA-like sequences in other closely related species of the Tetraonchidea and other monogeneans remain speculative.

### Phylogeny

**Fig. 4:**
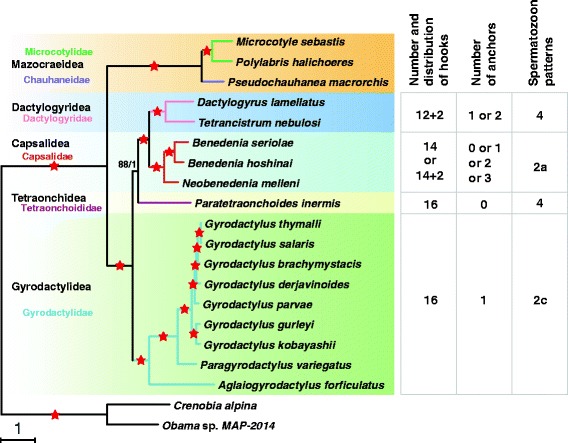
Phylogeny of the five orders inferred using concatenated amino acid sequences of 12 protein-coding genes. Scale-bar represents the estimated number of substitutions per site. Star symbol indicates that both methods produced the maximum statistical support value (BP = 100, BPP = 1.0), elsewhere both values are shown above the node as ML/BI. The number and distribution of hooks, number of anchors and spermatozoon patterns for the five orders are given to the right of the figure. The 14/12 + 2 in the first column refer to 14/12 marginal hooks +2 central hooks

Both phylogenetic analysis methods (BI and ML) produced phylograms with concordant branch topologies and high statistical support: all bootstrap support values were ≥ 88, and all Bayesian posterior probabilities were 1.0. Since both phylograms had the same topology, only the latter is shown (Fig. 4). Tree topology indicates the existence of two major clades: subclass Monopisthocotylea (Gyrodactylidea, Capsalidea, Tetraonchidea and Dactylogyridea) and subclass Polyopisthocotylea (Mazocraeidea). The Monopisthocotylea clade was further sub-divided into two clades, (Tetraonchidea + (Dactylogyridea + Capsalidea)) and Gyrodactylidea, both robustly supported (BP/BPP = 88/1 and 100/1, respectively).

Based on the topology obtained in our phylogenetic analysis, the order Tetraonchidea appears to be much more closely related to the Dactylogyridea + Capsalidea clade than to the order Gyrodactylidea. This result is not fully congruent with any of the previously proposed classifications [1–5, 56]. As the entire order Tetraonchidea was represented by a single species of the Tetraonchoididae (*P. inermis*) in our study, this topology should be interpreted with some caution. This relationship appears to be supported by spermiogenetic and spermatozoal ultrastructural characters [5] (Tetraonchidea and Dactylogyridea possess sperm pattern 4, exhibiting one axoneme; Fig. 4), but as the Gyrodactylidea and Capsalidea both possess two axonemes (patterns 2a and 2c), we can conclude that sperm morphology and mitochondrial phylogenomics produce incongruent signals. Our results also appear to reject the validity of taxonomic grouping of the Dactylogyridea, Tetraonchidea and Gyrodactylidea by the possession of 16 gyrodactylid-type marginal hooks, proposed by Bychowsky [1]. Similarly, the Tetraonchoididae were reassigned to the Gyrodactylidea by Boeger & Kritsky [4] mainly by hinged ‘gyrodactylid’ hooks, which is also incongruent with our results. Our results also do not support the basal phylogenetic position of the Capsalidea proposed by Boeger & Kritsky [4]. Finally, the results are also in disagreement with phylogenetic classifications based on molecular data: 18S ribosomal RNA sequences produced a topology in which capsalids were phylogenetically closer to gyrodactylids than dactylogyrids [11].

Although our phylogenetic framework failed to reach a consensus with any of the previous studies, either those based on morphological or on molecular data, it provides important new insights into the evolutionary history of the four monogenean orders, the Gyrodactylidea, Dactylogyridea, Capsalidea and Tetraonchidea. Morphological traits are often believed to exhibit a high frequency of homoplasy, especially in (parasitic) microscopic animals, whether as a consequence of subjective, or merely simplistic, definitions of a character state (artifact) [57], or of a convergent evolution caused by similar selection pressures on different taxonomic groups [58]. The existence of numerous incompatible phylogenetic hypotheses regarding DTCG group and the entire class Monogenea [1–7, 56] presents excellent proof that wrong conclusions are often reached when poorly-chosen or numerically insufficient morphological characters are invoked. Although molecular phylogenetics is a promising tool to address this issue [10, 11], future studies should rely on molecular markers that carry a sufficient amount of information to provide high phylogenetic resolution [12, 13].

### Gene order

**Fig. 5:**
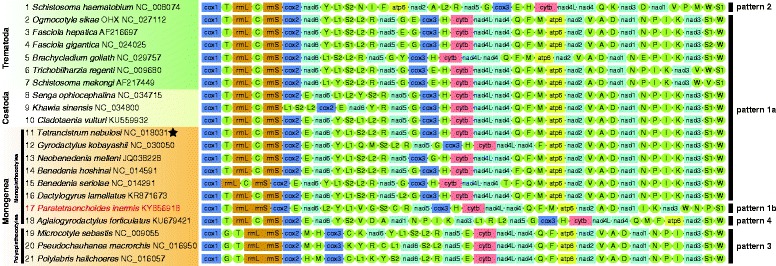
The 21 unique gene orders in neodermatan mitochondrial genomes filtered from 107 species. Representatives and the corresponding taxonomic category at the class/subclass level are shown on the left; star symbol denotes that the gene order is shared by Monogenea and Cestoda. Pattern types used here to classify gene orders are indicated on the right. Also, see Additional file 6: Figure S3)

*Paratetraonchoides inermis* exhibits an extensive reorganization of tRNAs in comparison to all other sequenced monogenean mt genomes (Fig. 5). However, disregarding the tRNA genes, the order of PCGs and rRNA genes within its mt genome conforms to the common neodermatan pattern [20, 59]. A gene order similarity matrix (Additional file 4: Table S3) based on all 36 genes also indicates that *P. inermis* was the most dissimilar among the compared monopisthocotylids, even including the unique order-possessing *A. forficulatus* [24]. The transformational pathway from *P. inermis* to the most similar gene arrangement, found in *T. nebulosi* and *Paragyrodactylus variegatus* (You, King, Ye & Cone, 2014), required two coupled transposition events, as well as three coupled long-range rearrangement operations, of which two were a TDRL, and one was a transposition (Additional file 5: Figure S2). Disregarding the two “non-standard” mt genomes, *P. inermis* and *A. forficulatus*, the remaining monopisthocotylids exhibit a remarkably conserved gene order [20, 23].

For a better comparison of gene order among neodermatans, we used MitoTool and iTOL to extract and visualize all available sequences for species of the Cestoda and Trematoda (Additional file 6: Figure S3) and filter out all non-unique gene orders. This resulted in a set of 21 unique gene orders: 11 for the Monogenea, four for the Cestoda (also see our recent publication [60]) and seven for the Trematoda (note that the eleventh gene order in Fig. 5 was shared by the Cestoda and Monogenea). This indicates an exceptional plasticity in the mitochondrial gene order in the Monogenea, as they merely represent 16.8% (18/107) of all available neodermatan mt genomes, but account for 52.4% (11/21) of all unique gene orders. In general, gene order within neodermatan mt genomes is relatively conserved: all of the Cestoda [60] and a majority of the Trematoda mt genomes exhibited only minor variations in the tRNA order (Fig. 5; pattern 1a). Exceptions were only the African/Indian schistosomes (pattern 2, with interchange of two gene blocks: *nad*5*-cox*3*-cytb-nad*4L*-nad*4 and *atp*6*-nad*2, and interchange of *nad*1 and *nad*3; Fig. 5) and the monogenean subtaxon Polyopisthocotylea (pattern 3). This was also observed by Webster et al. [19], but they did not have the latest two sequenced monopisthocotylid mitogenomes (*P. inermis* and *A. forficulatus*) at their disposal. The majority of Monopisthocotylidea species indeed do exhibit the most common gene order pattern 1a, but these two mt genomes both exhibit extensively altered gene orders: *A. forficulatus* exhibits pattern 4, with a transposal of an entire gene block [24]; and the newly sequenced *P. inermis* is the sole representative of the pattern 1b, exhibiting an extensive reshuffling of tRNA genes (Fig. 5).

We hypothesise that the most common pattern (1a) might be the primitive gene order (plesiomorphy) from which patterns 1b, 2, 3 and 4 were derived. This hypothesis is in agreement with previous studies [19, 21, 61], which considered the gene order of Asian schistosomatid mt genomes as the ancestral state, and the gene order of African/Indian schistosomatids a derived trait. Two non-standard gene patterns (4 and 1b) contradict the two hypotheses regarding the putative monogenean plesiomorphic gene arrangement *atp*6*-nad*2*-trnV-trnA-trnD-nad*1*-trnN-trnP-trnI* [23], and the discriminating markers between Monopisthocotylea and Polyopisthocotylea: *rrnL-trnC-rrnS* and *trnN-trnP-trnI-trnK* [48] (Table 3).

**Table 3:** Occurrence of gene blocks in the five proposed gene order patterns

Gene block/ Pattern	*cox1-T-rrnL*	*rrnL-C-rrnS*	*cox2-E-nad6*	*atp6-nad2-V-A-D-nad1*	*nad1-N-P-I-K-nad3*
Pattern 1a	Y^a^	Y	P	Y^b^	Y^c^
Pattern 1b	Y	–	Y	–	–
Pattern 2	Y	Y	–	–	–
Pattern 3	–	–	–	Y	–
Pattern 4	Y	Y	Y	–	Y

*Notes*: “Y” indicates that a gene block occurs in the corresponding pattern, whereas “–” indicates that the a gene block is broken up. “P” indicates that the gene block is present in all species exhibiting the pattern 1a, except for trematodes. “Y” with a superscript letter indicates the existence of minor exceptions in the gene block, wherein “Y^a^” denotes *Benedenia seriolae*; “Y^b^” denotes *Trichobilharzia regenti*, *Schistosoma mekongi* and *Schistosoma japonicum*; and “Y^d^” denotes *Brachycladium goliath*

Such rare gene arrangements are believed to be a promising tool for molecular systematics and phylogenetic reconstruction because mitochondrial gene order reversal events are very rare, and unique orders rarely occur independently in separate lineages [18]. However, the latest two sequenced mt genomes (*P. inermis* and *A. forficulatus*) show that monopisthocotylids do not possess a synapomorphic gene order, and instead suggest that gene order within this group may be evolving at uneven rates. This can create misleading evolutionary signals, which was observed before in some taxonomic groups [62–65]. Thus, while taking into consideration that our insight is curbed by the sparsity of available mt genomes, this finding provides a strong note of caution to researchers wishing to use gene order rearrangements as a tool for neodermatan phylogeny.

## Conclusions

Despite the limited availability of molecular data, our analysis provides three findings particularly worth noting. Firstly, there is no support for the sister-group relationship between the Gyrodactylidea and Tetraonchidea [1], nor for the allocation of the family Tetraonchoididae to Gyrodactylidea [4]. Instead, the Tetraonchidea exhibits a phylogenetic affinity with the Dactylogyridea + Capsalidea clade, which indirectly supports Lebedev’s traditional classification [2]. Secondly, the order Capsalidea is neither basal within the subclass Monopisthocotylea [4], nor forms a sister group with the Gyrodactylidea [10, 11]. Instead, it forms a sister clade with the Dactylogyridea, which is in full agreement with the two latest mitochondrial phylogenomic studies [24, 25] and lends further support to the traditional classifications by Bychowsky [1] and Lebedev [2]. Thirdly, the mitogenome of *P. inermis* provides several interesting findings from the genomic perspective as well: the unprecedentedly high A + T content of the entire genome and its elements, three tRNA-like sequences found in the mNCR, and a unique gene order. The latter indicates that gene order within monopisthocotylids may be evolving at uneven rates, thus creating misleading evolutionary signals. Heightened AT bias can confound phylogenetic inference [66] and the inclusion of only a handful of representatives for three orders (one for Tetraonchidea, two for Dactylogyridea and three for Capsalidea) in our analyses severely limits the phylogenetic resolution. Therefore, we are currently not able to generate a comprehensive phylogenetic hypothesis for the high-level phylogeny of Monopisthocotylea subclass, nor to conduct tests rigorous enough to be able to reject/accept with confidence the hypotheses put forward by the previous studies. Denser sampling and use of strategies alleviating potential compositional biases are needed to evaluate our phylogenetic results and resolve the phylogeny of monogeneans. Our work offsets the scarcity of molecular data for the order Tetraonchidea to some extent, providing a base both for the future fragmentary dataset studies (morphology data and single gene sequence-based molecular markers), as well as the future mitochondrial phylogenomics studies.

## Additional files

Additional file 1: Table S1. Primers used to amplify and sequence the mitochondrial genome of *Paratetraonchoides inermis*. NCR is non-coding region. (DOCX 16 kb)

Additional file 2: Table S2. The list of monogenean species and outgroups used for comparative mitogenomic and phylogenetic analyses. (XLSX 10 kb)

Additional file 3: Figure S1. Secondary structures of the three tRNA-like sequences found in the major noncoding region. (PDF 216 kb)

Additional file 4: Table S3. Pairwise comparison of 21 unique mitochondrial DNA gene orders among 107 neodermatan species. The analysis is based on the order of all 36 genes: 12 protein-coding, two rRNA and 22 tRNA genes. Taxa with identical gene order were removed and representative randomly chosen. Scores indicate the similarity between gene orders: the higher the score, the more similar the gene order, where “1254” represents an identical gene order. (XLSX 13 kb)

Additional file 5: Figure S2. Rearrangement pathway from *Paratetraonchoides inermis* to *Tetrancistrum nebulosi*. (PDF 294 kb)

Additional file 6: Figure S3. Gene orders of 107 neodermatan mitochondrial genomes. Identical gene orders are indicated (and numbered) by a vertical black line on the right. To facilitate the comparison and distance calculation in CREx program, we have re-annotated the mt genomes for which *trnS1* ^(AGN)^ *, S2* ^(UCN)^ *, L1* ^(CUN)^ and *L2* ^(UUR)^ were ambiguously annotated with the help of ARWEN and MitoTool programs. Mt. genomes for which it was impossible to produce a reliable and consistent annotation were not used in the analysis. In detail: no tRNAs could be predicted by ARWEN for positions 11,564 to 11,635 in *Orthocoelium streptocoelium* NC_028071; 11,604 to 11,667 of *Metorchis orientalis* NC_028008; 11,606 to 11,665 of *Fasciolopsis buski* NC_030528; 11,717 to 11,785 of *Fischoederius elongatus* NC_028001; and 7304 to 7362 of *Gastrothylax crumenifer* NC_027833; *rrnS* was absent from *Paragonimus westermani* NC_002354, *nad*2 was absent from *Fasciolopsis buski* KX449331, *nad*3 was absent from *Artyfechinostomum sufrartyfex* KX943545; a duplicated *trnS1* ^(AGN)^ was found in *Metagonimus yokogawai* NC_023249, and a duplicated *trnC* was found in *Schistosoma mansoni* NC_002545 and all the *Schistosoma japonicum* isolates. (PDF 4935 kb)

## Abbreviations

BI: Bayesian inference
BP: Bootstrap
BPP: Bayesian posterior probability
DTCG: Dactylogyridea, Tetraonchidea, Capsalidea and Gyrodactylidea
ML: Maximum likelihood
mNCR: Major non-coding region
PCGs: Protein-encoding genes
RSCU: Relative synonymous codon usage
TDRL: Tandem-duplication-random-loss

## References

[1] Bychowsky BE. [Monogenetic trematodes. Their systematics and phylogeny.] Moscow-Leningrad: Izdatel’stvo Akademiya Nauk SSSR. 1957: 509 pp (In Russian).

[2] Lebedev BI (1988). Monogenea in the light of new evidence and their position among platyhelminths. Angew Parasitol.

[3] Malmberg G (1990). On the ontogeny of the haptor and the evolution of the Monogenea. Syst Parasitol.

[4] Boeger WA, Kritsky DC (1993). Phylogeny and a revised classification of the Monogenoidea Bychowsky, 1937 (Platyhelminthes). Syst Parasitol.

[5] Justine J-L (1991). Cladistic study in the Monogenea (Platyhelminthes), based upon a parsimony analysis of spermiogenetic and spermatozoal ultrastructural characters. Int J Parasitol.

[6] Yamaguti S (1963). Systema helminthum. Vol. IV. Monogenea and Aspidocotylea.

[7] Llewellyn J (1970). Taxonomy, genetics and evolution of parasites. J Parasitol.

[8] Mollaret I, Jamieson BG, Justine J-L (2000). Phylogeny of the Monopisthocotylea and Polyopisthocotylea (Platyhelminthes) inferred from 28S rDNA sequences. Int J Parasitol.

[9] Bykhovsky BE, Gussev AV, Nagibina LF. [Monogenetic trematodes of the family Tetraonchoididae Bychowsky, 1951.] Tr Zool Inst Akad Nauk SSSR. 1965;35:140–66 (In Russian).

[10] Olson P, Littlewood D (2002). Phylogenetics of the Monogenea - evidence from a medley of molecules. Int J Parasitol.

[11] Šimková A, Plaisance L, Matějusová I, Morand S, Verneau O (2003). Phylogenetic relationships of the Dactylogyridae Bychowsky, 1933 (Monogenea: Dactylogyridea): the need for the systematic revision of the Ancyrocephalinae Bychowsky, 1937. Syst Parasitol.

[12] Huyse T, Buchmann K, Littlewood DTJ (2008). The mitochondrial genome of *Gyrodactylus derjavinoides* (Platyhelminthes: Monogenea) - a mitogenomic approach for *Gyrodactylus* species and strain identification. Gene.

[13] Delsuc F, Tsagkogeorga G, Lartillot N, Philippe H (2008). Additional molecular support for the new chordate phylogeny. Genesis.

[14] Shao R, Barker S (2007). Mitochondrial genomes of parasitic arthropods: implications for studies of population genetics and evolution. Parasitology.

[15] Perkins EM, Donnellan SC, Bertozzi T, Whittington ID (2010). Closing the mitochondrial circle on paraphyly of the Monogenea (Platyhelminthes) infers evolution in the diet of parasitic flatworms. Int J Parasitol.

[16] Park JK, Kim KH, Kang S, Kim W, Eom KS, Littlewood DTJ (2007). A common origin of complex life cycles in parasitic flatworms: evidence from the complete mitochondrial genome of *Microcotyle sebastis* (Monogenea: Platyhelminthes). BMC Evol Biol.

[17] Rokas A, Holland PWH (2000). Rare genomic changes as a tool for phylogenetics. Trends Ecol Evol.

[18] Boore JL, Fuerstenberg SI (2008). Beyond linear sequence comparisons: the use of genome-level characters for phylogenetic reconstruction. Philos Trans R Soc Lond Ser B Biol Sci.

[19] Webster BL, Littlewood DTJ (2012). Mitochondrial gene order change in *Schistosoma* (Platyhelminthes: Digenea: Schistosomatidae). Int J Parasitol.

[20] Wey-Fabrizius AR, Podsiadlowski L, Herlyn H, Hankeln T (2013). Platyzoan mitochondrial genomes. Mol Phylogenet Evol.

[21] Littlewood DTJ, Lockyer AE, Webster BL, Johnston DA, Le TH (2006). The complete mitochondrial genomes of *Schistosoma haematobium* and *Schistosoma spindale* and the evolutionary history of mitochondrial genome changes among parasitic flatworms. Mol Phylogenet Evol.

[22] Zhang J, Wu X, Xie M, Xu X, Li A (2011). The mitochondrial genome of *Polylabris halichoeres* (Monogenea: Microcotylidae). Mitochondrial DNA.

[23] Zhang J, Wu X, Xie M, Li A (2012). The complete mitochondrial genome of *Pseudochauhanea macrorchis* (Monogenea: Chauhaneidae) revealed a highly repetitive region and a gene rearrangement hot spot in Polyopisthocotylea. Mol Biol Rep.

[24] Bachmann L, Fromm B, de Azambuja LP, Boeger WA (2016). The mitochondrial genome of the egg-laying flatworm *Aglaiogyrodactylus forficulatus* (Platyhelminthes: Monogenoidea). Parasit Vectors.

[25] Zhang D, Zou H, Wu SG, Li M, Jakovlic I, Zhang J, Chen R, et al. Sequencing, characterization and phylogenomics of the complete mitochondrial genome of *Dactylogyrus lamellatus* (Monogenea: Dactylogyridae). J Helminthol. 2017:1–12. 10.1017/S0022149X17000578 28660842

[26] Altschul SF, Gish W, Miller W, Myers EW, Lipman DJ (1990). Basic local alignment search tool. J Mol Biol.

[27] Katoh K, Standley DM (2013). MAFFT multiple sequence alignment software version 7: improvements in performance and usability. Mol Biol Evol.

[28] Kearse M, Moir R, Wilson A, Stones-Havas S, Cheung M, Sturrock S, Buxton S (2012). Geneious Basic: an integrated and extendable desktop software platform for the organization and analysis of sequence data. Bioinformatics.

[29] Laslett D, Canback B (2008). ARWEN: a program to detect tRNA genes in metazoan mitochondrial nucleotide sequences. Bioinformatics.

[30] Wyman SK, Jansen RK, Boore JL (2004). Automatic annotation of organellar genomes with DOGMA. Bioinformatics.

[31] Bernt M, Donath A, Juhling F, Externbrink F, Florentz C, Fritzsch G, Putz J (2013). MITOS: improved *de novo* metazoan mitochondrial genome annotation. Mol Phylogenet Evol.

[32] Zhang D (2016). MitoTool software.

[33] Hadley W (2010). ggplot2: Elegant graphics for data analysis. J Stat Softw.

[34] Tamura K, Peterson D, Peterson N, Stecher G, Nei M, Kumar S. MEGA5: Molecular Evolutionary Genetics Analysis using maximum likelihood, evolutionary distance, and maximum parsimony methods. Mol Biol Evol. 2011;28(10):2731–9. 10.1093/molbev/msr121 PMC3203626 21546353

[35] Zhang D. rscuStack package. 2017. https://github.com/dongzhang0725/Python_mtDNA/tree/master/rscuStack. Accessed 17 June 2017.

[36] Bernt M, Merkle D, Ramsch K, Fritzsch G, Perseke M, Bernhard D, Schlegel M (2007). CREx: inferring genomic rearrangements based on common intervals. Bioinformatics.

[37] Zhang D (2016). BioSuite software.

[38] Abascal F, Zardoya R, Posada D (2005). ProtTest: selection of best-fit models of protein evolution. Bioinformatics.

[39] Silvestro D, Michalak I (2011). raxmlGUI: a graphical front-end for RAxML. Org Divers Evol.

[40] Ronquist F, Teslenko M, van der Mark P, Ayres DL, Darling A, Höhna S, Larget B (2012). MrBayes 3.2: efficient Bayesian phylogenetic inference and model choice across a large model space. Syst Biol.

[41] Letunic I, Bork P (2016). Interactive tree of life (iTOL) v3: an online tool for the display and annotation of phylogenetic and other trees. Nucleic Acids Res.

[42] Le TH BD, DP MM (2002). Mitochondrial genomes of parasitic flatworms. Trends Parasitol.

[43] Salvato P, Simonato M, Battisti A, Negrisolo E (2008). The complete mitochondrial genome of the bag-shelter moth *Ochrogaster lunifer* (Lepidoptera, Notodontidae). BMC Genomics.

[44] Chen HX, Sun SC, Sundberg P, Ren WC, Norenburg JL (2012). A comparative study of nemertean complete mitochondrial genomes, including two new ones for *Nectonemertes* cf. *mirabilis* and *Zygeupolia rubens*, may elucidate the fundamental pattern for the phylum Nemertea. BMC Genomics.

[45] Zhang J, Wu X, Li Y, Zhao M, Xie M, Li A (2014). The complete mitochondrial genome of *Neobenedenia melleni* (Platyhelminthes: Monogenea): mitochondrial gene content, arrangement and composition compared with two *Benedenia* species. Mol Biol Rep.

[46] Huyse T, Plaisance L, Webster BL, Mo TA, Bakke TA, Bachmann L, Littlewood DTJ (2007). The mitochondrial genome of *Gyrodactylus salaris* (Platyhelminthes: Monogenea), a pathogen of Atlantic salmon (*Salmo salar*). Parasitology.

[47] Plaisance L, Huyse T, Littlewood DTJ, Bakke TA, Bachmann L (2007). The complete mitochondrial DNA sequence of the monogenean *Gyrodactylus thymalli* (Platyhelminthes: Monogenea), a parasite of grayling (*Thymallus thymallus*). Mol Biochem Parasitol.

[48] Ye F, King SD, Cone DK, You P (2014). The mitochondrial genome of *Paragyrodactylus variegatus* (Platyhelminthes: Monogenea): differences in major non-coding region and gene order compared to *Gyrodactylus*. Parasit Vectors.

[49] Ye F, Easy RH, King SD, Cone DK, You P (2016). Comparative analyses within *Gyrodactylus* (Platyhelminthes: Monogenea) mitochondrial genomes and conserved polymerase chain reaction primers for gyrodactylid mitochondrial DNA. J Fish Dis.

[50] Zou H, Zhang D, Li WX, Zhou S, Wu SG, Wang GT (2016). The complete mitochondrial genome of *Gyrodactylus gurleyi* (Platyhelminthes: Monogenea). Mitochondrial DNA Part B..

[51] Zhang D, Zou H, Zhou S, Wu SG, Li WX, Wang GT (2016). The complete mitochondrial genome of *Gyrodactylus kobayashii* (Platyhelminthes: Monogenea). Mitochondrial DNA Part B.

[52] Zhang J, Wu X, Li Y, Xie M, Li A (2014). The complete mitochondrial genome of *Tetrancistrum nebulosi* (Monogenea: Ancyrocephalidae). Mitochondrial DNA.

[53] Kim MJ, Kang AR, Jeong HC, Kim KG, Kim I (2011). Reconstructing intraordinal relationships in Lepidoptera using mitochondrial genome data with the description of two newly sequenced lycaenids, *Spindasis takanonis* and *Protantigius superans* (Lepidoptera: Lycaenidae). Mol Phylogenet Evol.

[54] Park JS, Kim MJ, Jeong SY, Kim SS, Kim I (2016). Complete mitochondrial genomes of two gelechioids, *Mesophleps albilinella* and *Dichomeris ustalella* (Lepidoptera: Gelechiidae), with a description of gene rearrangement in Lepidoptera. Curr Genet.

[55] Cameron SL (2014). Insect mitochondrial genomics: implications for evolution and phylogeny. Annu Rev Entomol.

[56] Lambert A (1980). Oncomiracidiums et phylogénèse des Monogènes (Plathelminthes), 2ème partie: Structures argyrophiles des oncomiracidiums et phylogénèse des Monogenea. Ann Parasitol Hum Comp.

[57] Perkins EM, Donnellan SC, Bertozzi T, Chisholm LA, Whittington ID (2009). Looks can deceive: molecular phylogeny of a family of flatworm ectoparasites (Monogenea: Capsalidae) does not reflect current morphological classification. Mol Phylogenet Evol.

[58] Poulin R, Morand S (2000). The diversity of parasites. Q Rev Biol.

[59] Egger B, Bachmann L, Fromm B (2017). *Atp8* is in the ground pattern of flatworm mitochondrial genomes. BMC Genomics.

[60] Li WX, Zhang D, Boyce K, Xi BW, Zou H, Wu SG, Li M (2017). The complete mitochondrial DNA of three monozoic tapeworms in the Caryophyllidea: a mitogenomic perspective on the phylogeny of eucestodes. Parasit Vectors.

[61] Wang Y, Wang CR, Zhao GH, Gao JF, Li MW, Zhu XQ (2011). The complete mitochondrial genome of *Orientobilharzia turkestanicum* supports its affinity with African *Schistosoma* spp. Infect Genet Evol.

[62] Babbucci M, Basso A, Scupola A, Patarnello T, Negrisolo E (2014). Is it an ant or a butterfly? Convergent evolution in the mitochondrial gene order of Hymenoptera and Lepidoptera. Genome Biol Evol.

[63] Dowton M, Austin AD (1999). Evolutionary dynamics of a mitochondrial rearrangement “hot spot” in the Hymenoptera. Mol Biol Evol.

[64] San MD (2005). A hotspot of gene order rearrangement by tandem duplication and random loss in the vertebrate mitochondrial genome. Mol Biol Evol.

[65] Wang J-G, Zhang D, Jakovlić I, Wang W-M. Sequencing of the complete mitochondrial genomes of eight freshwater snail species exposes pervasive paraphyly within the Viviparidae family (Caenogastropoda). PLoS One. 2017; doi:10.1371/journal.pone.0181699. 10.1371/journal.pone.0181699 PMC5526530 28742843

[66] Talavera G, Vila R (2011). What is the phylogenetic signal limit from mitogenomes? The reconciliation between mitochondrial and nuclear data in the Insecta class phylogeny. BMC Evol Biol.

