# Supplementary material for: Sequencing of the complete mitochondrial genome of a fish-parasitic flatworm Paratetraonchoides inermis (Platyhelminthes: Monogenea): tRNA gene arrangement reshuffling and implications for phylogeny
Source: Parasit Vectors. 2017 Oct 10;10:462. doi: 10.1186/s13071-017-2404-1 (PMC5633893; doi:10.1186/s13071-017-2404-1)
Supplement: Supplementary file 1 — Primers used to amplify and sequence the mitochondrial genome of Paratetraonchoides inermis. NCR is non-coding region. (DOCX 16 kb) [file 13071_2017_2404_MOESM1_ESM.docx]

**Additional file 1: Table S1.** Primers used to amplify and sequence the mitochondrial genome of *Paratetraonchoides inermis*. NCR is non-coding region.

| **Fragment**  **No.** | **Gene or**  **region** | **Primer**  **name** | **Sequence (5’-3’)** | **Length**  **(bp)** |
| --- | --- | --- | --- | --- |
| F1 | NCR | XZF1 | GAAGATTTATATTAGTGTAAGAC | 483 |
|  |  | XZR1 | GAATATCTTTATAGCCATAAAAC |  |
| F2 | NCR*-cox3* | XZF2 | CACACTAATATGAATTTTC | 802 |
|  |  | XZR2 | CAAAAACTATCAATATAATG |  |
| F3 | *cox3* | XZF3 | CTTTCTTAATAATGATAC | 414 |
|  |  | XZR3 | TACTACAATCTCTAAACTC |  |
| F4 | *cox3* | XZF4 | GAGHTHCCDTTTYTTGGBTG | 334 |
|  |  | XZR4 | CDACAAARTGTCARTATCA |  |
| F5 | *cox3-nad1* | XZF5 | GTTGTTTTTGTACTGTTGGG | 4852 |
|  |  | XZR5 | AGTATACTCCAATCATACC |  |
| F6 | *nad1* | XZF6 | CGKAAGGGNCCTAAHAAGGTTGG | 704 |
|  |  | XZR6 | CGAAHTCGHGGTARAGADGCACG |  |
| F7 | *nad1-cox1* | XZF7 | CCATTTGATTATGCTGAATC | 2058 |
|  |  | XZR7 | CCCATATAACCAAAAACTTCATC |  |
| F8 | *cox1* | XZF8 | GATCCDAGRGGAGGWGGTGATC | 666 |
|  |  | XZR8 | ACAAACWCGACGWGGTAATCC |  |
| F9 | *cox1-cox2* | XZF9 | TCCTTACTTTGATGATGACC | 2558 |
|  |  | XZR9 | GTCCTGGAACAGCATCTATC |  |
| F10 | *cox2* | XZF10 | TAGTDGGACGACARTGATACTG | 271 |
|  |  | XZR10 | CCACATAARTCTCTACARTAACC |  |
| F11 | *cox2-nad5* | XZF11 | GATAGATGCTGTTCCAGGAC | 1887 |
|  |  | XZR11 | CTCCTGCAGCAACTAAAGTAG |  |
| F12 | *nad5* | XZF12 | GARGCTATGCGDGCTCCAACACC | 470 |
|  |  | XZR12 | TGCTTAGAAAARAATATACC |  |
| F13 | *nad5-*NCR | XZF13 | CTCATGGTATAATTAAGTGTG | 1263 |
|  |  | XZR13 | ACTCAATGCCATATTTATTG |  |
