# Supplementary figures and images for: Sequencing of the complete mitochondrial genome of a fish-parasitic flatworm Paratetraonchoides inermis (Platyhelminthes: Monogenea): tRNA gene arrangement reshuffling and implications for phylogeny
Source: Parasit Vectors. 2017 Oct 10;10:462. doi: 10.1186/s13071-017-2404-1 (PMC5633893; doi:10.1186/s13071-017-2404-1)

*Paratetraonchoides inermis*

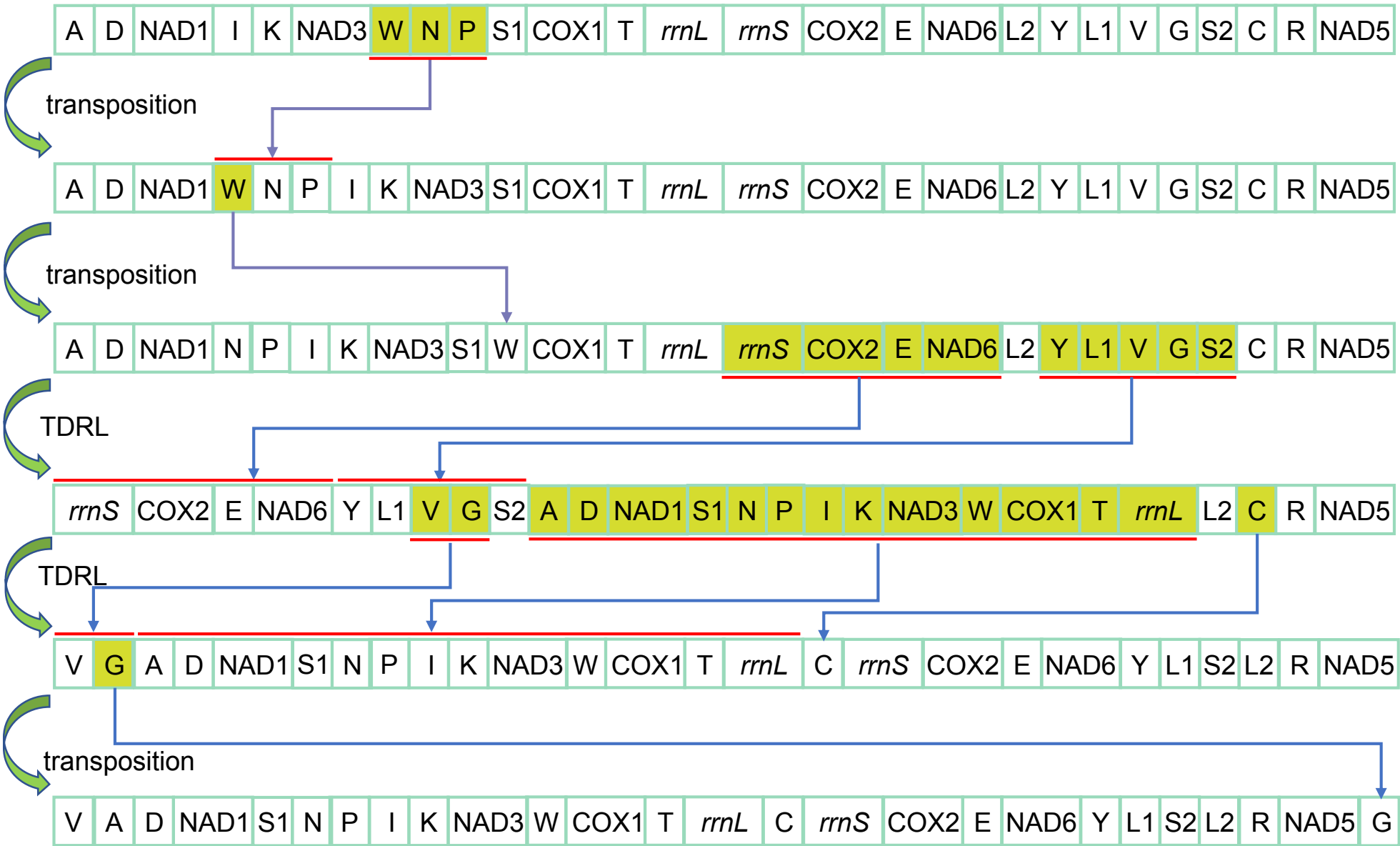

*Tetrancistrum nebulosi*

Supplement: Supplementary file 5 — Rearrangement pathway from Paratetraonchoides inermis to Tetrancistrum nebulosi. (PDF 294 kb) [file 13071_2017_2404_MOESM5_ESM.pdf]
