# Supplementary material for: Sequencing of the complete mitochondrial genome of a fish-parasitic flatworm Paratetraonchoides inermis (Platyhelminthes: Monogenea): tRNA gene arrangement reshuffling and implications for phylogeny
Source: Parasit Vectors. 2017 Oct 10;10:462. doi: 10.1186/s13071-017-2404-1 (PMC5633893; doi:10.1186/s13071-017-2404-1)
Supplement: Supplementary file 6 — Gene orders of 107 neodermatan mitochondrial genomes. Identical gene orders are indicated (and numbered) by a vertical black line on the right. To facilitate the comparison and distance calculation in CREx program, we have re-annotated the mt genomes for which trnS1 (AGN) , S2 (UCN) , L1 (CUN) and L2 (UUR) were ambiguously annotated with the help of ARWEN and MitoTool programs. Mt. genomes for which it was impossible to produce a reliable and consistent annotation were not used in the analysis. In detail: no tRNAs could be predicted by ARWEN for positions 11,564 to 11,635 in Orthocoelium streptocoelium NC_028071; 11,604 to 11,667 of Metorchis orientalis NC_028008; 11,606 to 11,665 of Fasciolopsis buski NC_030528; 11,717 to 11,785 of Fischoederius elongatus NC_028001; and 7304 to 7362 of Gastrothylax crumenifer NC_027833; rrnS was absent from Paragonimus westermani NC_002354, nad2 was absent from Fasciolopsis buski KX449331, nad3 was absent from Artyfechinostomum sufrartyfex KX943545; a duplicated trnS1 (AGN) was found in Metagonimus yokogawai NC_023249, and a duplicated trnC was found in Schistosoma mansoni NC_002545 and all the Schistosoma japonicum isolates. (PDF 4935 kb) [file 13071_2017_2404_MOESM6_ESM.pdf]

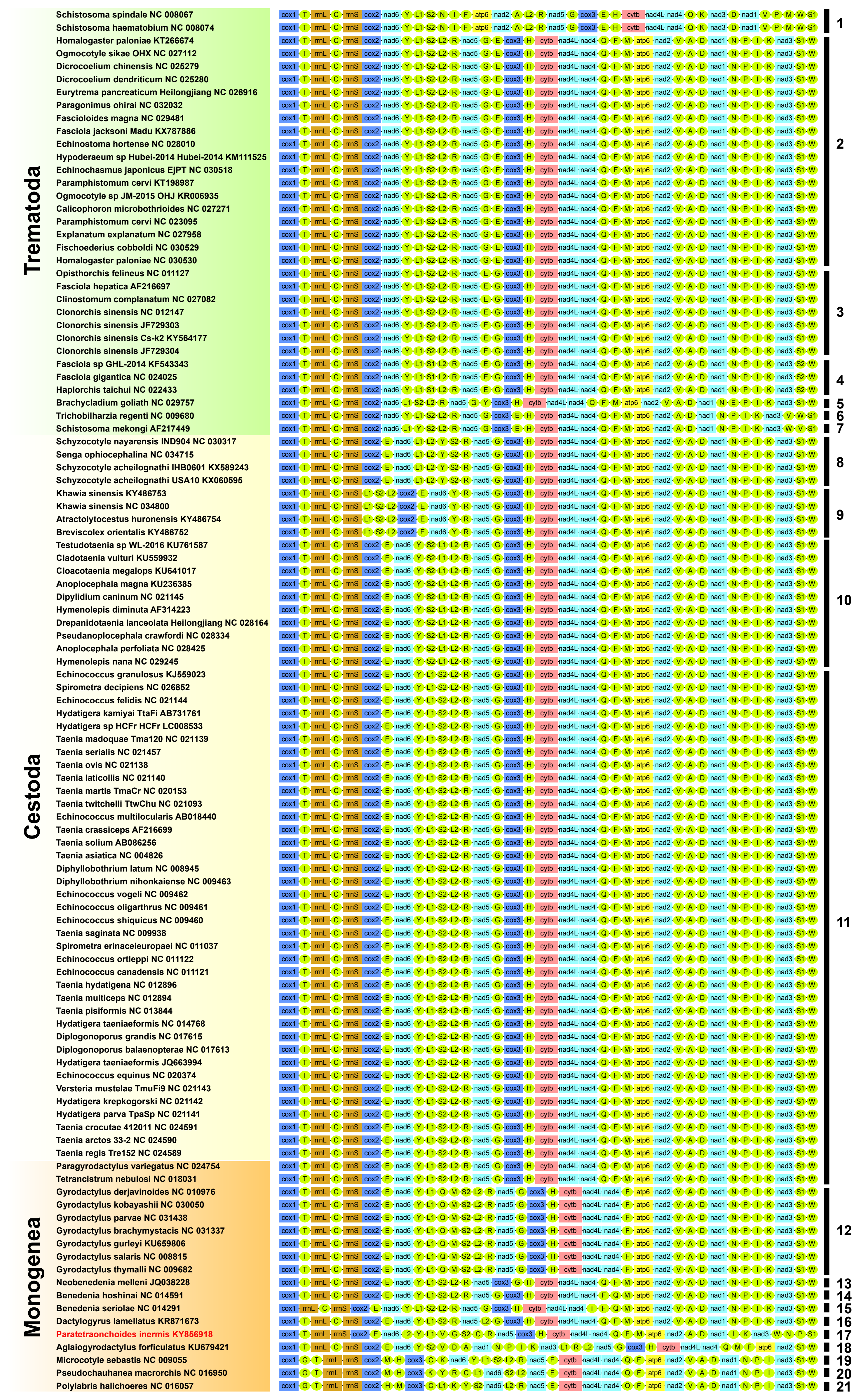
Trematoda
Schistosoma spindale NC 008067
Schistosoma haematobium NC 008074
Homalogaster paloniae KT266674
Ogmocotyle sikae OHX NC 027112
Dicrocoelium chinensis NC 025279
Dicrocoelium dendriticum NC 025280
Eurytrema pancreaticum Heilongjiang NC 026916
Paragonimus ohirai NC 032032
Fascioloides magna NC 029481
Fasciola jacksoni Madu KX787886
Echinostoma hortense NC 028010
Hypoderaeum sp Hubei-2014 Hubei-2014 KM111525
Echinochasmus japonicus EjPT NC 030518
Paramphistomum cervi KT198987
Ogmocotyle sp JM-2015 OHJ KR006935
Calicophoron microbothrioides NC 027271
Paramphistomum cervi NC 023095
Explanatum explanatum NC 027958
Fischoederius cobboldi NC 030529
Homalogaster paloniae NC 030530
Opisthorchis felineus NC 011127
Fasciola hepatica AF216697
Clinostomum complanatum NC 027082
Clonorchis sinensis NC 012147
Clonorchis sinensis JF729303
Clonorchis sinensis Cs-k2 KY564177
Clonorchis sinensis JF729304
Fasciola sp GHL-2014 KF543343
Fasciola gigantica NC 024025
Haplorchis taichui NC 022433
Brachycladium goliath NC 029757
Trichobilharzia regenti NC 009680
Schistosoma mekongi AF217449
Cestoda
Schyzocotyle nayarensis IND904 NC 030317
Senga ophiocephalina NC 034715
Schyzocotyle acheilognathi IHB0601 KX589243
Schyzocotyle acheilognathi USA10 KX060595
Khawia sinensis KY486753
Khawia sinensis NC 034800
Atractolytocestus huronensis KY486754
Breviscolex orientalis KY486752
Testudotaenia sp WL-2016 KU761587
Cladotaenia vulturi KU559932
Cloacotaenia megalops KU641017
Anoplocephala magna KU236385
Dipylidium caninum NC 021145
Hymenolepis diminuta AF314223
Drepanidotaenia lanceolata Heilongjiang NC 028164
Pseudanoplocephala crawfordi NC 028334
Anoplocephala perfoliata NC 028425
Hymenolepis nana NC 029245
Echinococcus granulosus KJ559023
Spirometra decipiens NC 026852
Echinococcus felidis NC 021144
Hydatigera kamiyai TtaFi AB731761
Hydatigera sp HCFr HCFr LC008533
Taenia madoquae Tma120 NC 021139
Taenia serialis NC 021457
Taenia ovis NC 021138
Taenia laticollis NC 021140
Taenia martis TmaCr NC 020153
Taenia twitchelli TtwChu NC 021093
Echinococcus multilocularis AB018440
Taenia crassiceps AF216699
Taenia solium AB086256
Taenia asiatica NC 004826
Diphyllobothrium latum NC 008945
Diphyllobothrium nihonkaiense NC 009463
Echinococcus vogeli NC 009462
Echinococcus oligarthrus NC 009461
Echinococcus shiquicus NC 009460
Taenia saginata NC 009938
Spirometra erinaceieuropaei NC 011037
Echinococcus ortleppi NC 011122
Echinococcus canadensis NC 011121
Taenia hydatigena NC 012896
Taenia multiceps NC 012894
Taenia pisiformis NC 013844
Hydatigera taeniaeformis NC 014768
Diplogonoporus grandis NC 017615
Diplogonoporus balaenopterae NC 017613
Hydatigera taeniaeformis JQ663994
Echinococcus equinus NC 020374
Versteria mustelae TmuFi9 NC 021143
Hydatigera krepkogorski NC 021142
Hydatigera parva TpaSp NC 021141
Taenia crocutae 412011 NC 024591
Taenia arctos 33-2 NC 024590
Taenia regis Tre152 NC 024589
Monogenea
Paragyrodactylus variegatus NC 024754
Tetrancistrum nebulosi NC 018031
Gyrodactylus derjavinoides NC 010976
Gyrodactylus kobayashii NC 030050
Gyrodactylus parvae NC 031438
Gyrodactylus brachymystacis NC 031337
Gyrodactylus gurleyi KU659806
Gyrodactylus salaris NC 008815
Gyrodactylus thymalli NC 009682
Neobenedenia melleni JQ038228
Benedenia hoshinai NC 014591
Benedenia seriolae NC 014291
Dactylogyrus lamellatus KR871673
Paratetraonchoides inermis KY856918
Aglaiogyrodactylus forficulatus KU679421
Microcotyle sebastis NC 009055
Pseudochauhanea macrorchis NC 016950
Polylabris halichoeres NC 016057
1
2
3
4
5
6
7
8
9
10
11
12
13
14
15
16
17
18
19
20
21
