# Supplementary material for: Sequencing of the complete mitochondrial genome of a fish-parasitic flatworm Paratetraonchoides inermis (Platyhelminthes: Monogenea): tRNA gene arrangement reshuffling and implications for phylogeny
Source: Parasit Vectors. 2017 Oct 10;10:462. doi: 10.1186/s13071-017-2404-1 (PMC5633893; doi:10.1186/s13071-017-2404-1)
Supplement: Supplementary file 3 — Secondary structures of the three tRNA-like sequences found in the major noncoding region. (PDF 216 kb) [file 13071_2017_2404_MOESM3_ESM.pdf]

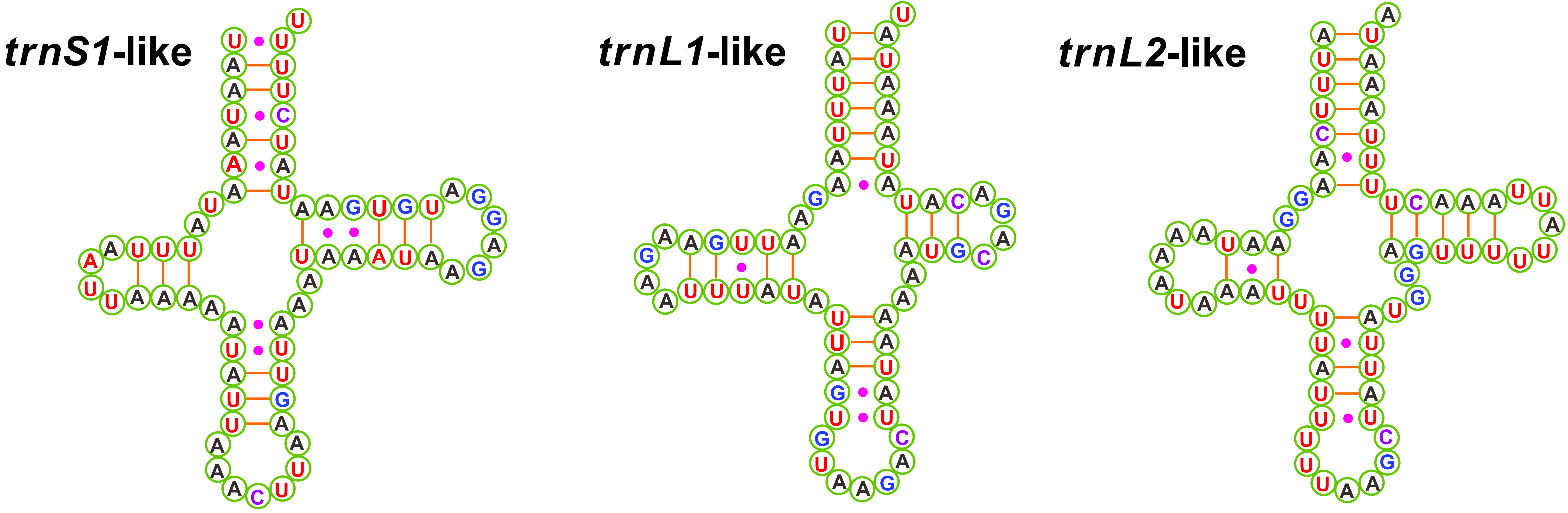
trnS1-like
trnL1-like
trnL2-like
